# A feasibility study of a randomised controlled trial to examine the impact of the ABCDE bundle on quality of life in ICU survivors

**DOI:** 10.1186/s40814-017-0224-x

**Published:** 2018-01-11

**Authors:** Kellie Sosnowski, Marion L. Mitchell, Hayden White, Lynette Morrison, Joanne Sutton, Jessica Sharratt, Frances Lin

**Affiliations:** 1 Intensive Care Unit, Logan Hospital, Logan City, Australia; 2 Griffith University, Brisbane, Australia; 3 School of Nursing and Midwifery, Menzies Health Institute Queensland, Griffith University, Gold Coast, Australia; 4 Intensive Care Unit, Princess Alexandra Hospital, Brisbane, Australia

**Keywords:** ABCDE bundle, Critical illness, Delirium, Muscle weakness, Quality of life, Rehabilitation, Intensive care

## Abstract

**Background:**

Early rehabilitation has been found to prevent delirium and weakness that can hamper the recovery of intensive care unit (ICU) survivors. Integrated clinical practice guidelines for managing patient pain, agitation and delirium (PAD) have been developed. The Awakening and Breathing Coordination, Delirium monitoring/management, and Early exercise/mobility (ABCDE) bundle provides a strategy to implement PAD guidelines into everyday clinical practice. However, there is limited evidence on the effectiveness of the ABCDE bundle in the literature.

The purpose of this study was to evaluate the feasibility of conducting a full-scale randomised controlled trial comparing the ABCDE bundle to standard care in an ICU. Trial feasibility was defined as the successful recruitment and retention of trial participants, adherence to the intervention, identification of barriers to the intervention, and the rigorous collection of outcome data.

**Methods:**

A prospective, single-centre, randomised controlled feasibility study was conducted. Thirty adult mechanically ventilated participants were recruited from an eight-bed ICU in south east Queensland, Australia, between April 2015 and December 2015. Participants were randomised to receive either the ABCDE bundle or standard routine management. The ABCDE bundle integrated prescribed awakening and breathing trials, delirium monitoring and management, and prescribed exercise and mobility regimes. Feasibility outcomes measured included recruitment and retention rates, intervention fidelity, and the feasibility of participant outcome data collection. Outcome measurement assessors were blinded to participant assignment. It was not possible to blind the research team or the participant to group assignment.

**Results:**

In total, 30 (81.1%) of 37 eligible participants consented and were randomised to the intervention group (*n* = 15) or the control group (*n* = 15). Of these, 23 (76.6%) participants successfully completed the 90-day post discharge assessment. A lengthy recruitment period of 8 months was related to overly stringent inclusion and exclusion criteria. Intervention adherence exceeded defined success rates with participation in awakening and breathing trials, delirium monitoring and exercise interventions performed on 80.2, 97.4 and 90.2% of ventilated days respectively. Outcome assessments were successfully and accurately performed at ICU and hospital discharge and 90-day post hospital discharge. Intervention participants were deemed to be delirious on 39.6% of mechanically ventilated days indicating a requirement for a scripted regime to prevent delirium.

**Conclusions:**

With minor adjustment of inclusion and exclusion criteria, the inclusion of delirium management protocols, and encouragement of family engagement and involvement, a large-scale definitive randomised controlled trial to test the impact of the ABCDEF bundle will be feasible.

**Trial registration:**

Australian New Zealand Clinical Trials Registry 12614000763640 Date registered 17/08/2014

**Electronic supplementary material:**

The online version of this article (10.1186/s40814-017-0224-x) contains supplementary material, which is available to authorized users.

## Background

### Introduction

Critically ill patients commonly suffer profound weakness, pain, and delirium which are inextricably linked [1]. Limited physical function and adverse effects associated with immobilisation are likely to worsen if cognition is not intact [2]. Physical impairments related to these issues may last for years after discharge from the intensive care unit (ICU) [3].

Integrated pain, agitation and delirium (PAD) clinical practice guidelines for critically ill patients using pharmacologic and non-pharmacologic approaches have been developed following rigorous research and aim to prevent long-term detrimental patient outcomes [4]. To date, these approaches have not necessarily produced reductions in negative sequela for the critically ill [5]. Researchers agree that delirium and ICU-associated weakness (ICUAW) remain pervasive and under-recognised within our ICUs [6].

The ABCDE bundle focusses on a way to implement the PAD guidelines to reduce delirium and weakness related to over-sedation, prolonged mechanical ventilation and forced immobility in mechanically ventilated critically ill patients [7]. This bundle of cares as described by Balas and colleagues [7] comprises three inter-related components: coordination of awakening and breathing trials, monitoring and management of delirium, and early exercise. Clinicians are increasingly incorporating the ABCDE bundle into clinical practice [8] and investigating outcomes associated with its use in the ICU [9].

A prospective before–after study tested the effectiveness and safety of incorporating the ABCDE bundle into everyday practice [10]. Balas and colleagues [10] recruited 296 patients (146 in the pre-group and 150 in the post bundle implementation group) from five adult ICUs, a step-down unit, and an oncology special care unit in a large tertiary hospital in the USA. The authors found a number of significant patient benefits that resulted from incorporating the ABCDE bundle including decreased duration of ventilation, reduced delirium and increased episodes of mobilisation [10].

Although results of this study were promising, limitations of the study design warrant consideration. Balas and colleagues [10] acknowledged that education regarding the ABCDE protocol applied during the pre-implementation period may have influenced study results. There may have been contamination during the pre-test period with inadvertent implementation of aspects of the intervention. As with all pre-post studies, the effect of history on the internal validity cannot be disregarded. Rigorous RCTs are therefore required to adequately test the effect of this bundle. To the best of our knowledge, there are no published RCTs examining the ABCDE bundle and its impact on ICU survivor quality of life.

### Objectives

In this study, we aimed to test the feasibility of conducting an RCT to examine the impact of the ABCDE bundle on quality of life in ICU survivors.

Our research question was: What is the feasibility of conducting a RCT using the ABCDE bundle compared with usual practice to improve ICU survivors’ quality of life? Feasibility outcomes included successful recruitment and retention of trial participants, intervention fidelity, identification of barriers to implementation of the intervention, and the feasibility of collecting outcome assessment data. The CONSORT extension statement checklist for pilot studies [11] was used as a guide to ensure complete and transparent reporting of our study (See Additional file 1).

## Methods

### Trial design

A prospective, single-centre, single-blinded, equally randomised (1:1), controlled feasibility study was conducted between April 2015 and March 2016 in an eight-bed, level two [12] ICU in south east Queensland, Australia. The protocol for this pilot trial is available on the Australian New Zealand Clinical Trials Registry website.

We were cognisant that a study involving the multicomponent ABCDE bundle is complex and that many trials involving complex interventions fail to prove a significant positive result [13]. This may be related to poor implementation of the intervention or substandard study design rather than genuine ineffectiveness. To ensure the effectiveness of our study protocol, we have utilised the principles of the United Kingdom Medical Research Council’s framework for the design and evaluation of complex health care intervention to prepare for a rigorous and appropriately powered future RCT [14].

The initial development phase of the framework required a comprehensive review of pertinent literature relating to the early rehabilitation of critically ill patients [15]. Our integrative review informed the modelling component of the framework which involved introducing the ABCDE bundle of cares into an ICU as detailed in the following method section. The results of the feasibility and piloting phase of the framework are presented in this manuscript. The outcome of this trial will determine if and how we can progress to an RCT to compare the ABCDE bundle with standard ICU practice.

### Participants

Schweickert et al. [2] had reported improved functional outcomes in patients who had received physical therapy within 72 h of the initiation of mechanical ventilation. Thus, patients admitted to the ICU were included if they were aged over 18 years, had been mechanically ventilated for 48 h and were expected to require ventilation for at least a further 24 h.

Patients were excluded from the study if a premorbid functional or cognitive impairment precluded engagement in exercise. Exclusion criteria included an inability to mobilise 3 meters before the current ICU illness; were diagnosed with neuromuscular disease that could impair ventilator weaning; had suffered an acute stroke; were not for active resuscitation; had been readmitted to ICU within the current hospitalisation; or were not expected to survive the current ICU admission.

During the week, potential participants were identified during the 8 am ward round by a member of the research nursing team. Senior medical or nursing staff contacted the on-call member of the research team on the week-end if a suitable participant was identified.

Consent was initially provided by the substitute decision-maker as the participant lacked capacity related to altered consciousness, sedation and medical condition. Once able, the participant was asked to provide deferred consent to continue in the study.

**Fig. 1:**
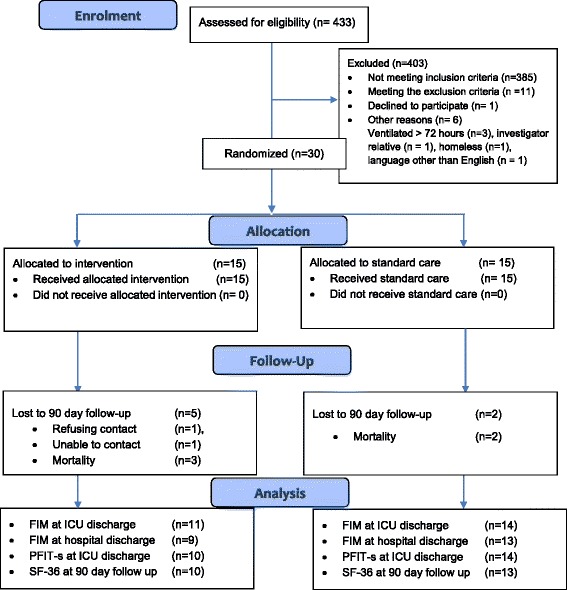
Flow of study participants

Patient flow through the research process is depicted in the CONSORT flow diagram (Fig. 1).

### Sample size

Thirty participants were recruited with 15 in each group. This number was consistent with recommendations for pilot and feasibility studies where samples of 10 to 20 participants per group have been deemed adequate to assess feasibility outcomes [16, 17].

### Randomisation

Participants were randomised without stratification to either the intervention or control group using a computer-generated allocation sequence using permuted blocks of two (www. randomizer.org). The assigned group allocations were concealed in opaque (when held to the light) envelopes that were consecutively numbered and sealed. The envelopes were prepared by personnel not connected to the study and were stored in a secured office within the ICU. An envelope was opened by a member of the research team, and a participant was assigned to the allocated group once eligibility criteria were met and informed consent obtained.

### Blinding

It was not possible to blind the research team or the participant to group assignment. However, participant outcome measurement assessors were blinded to participant assignment as they were separate to the health care providers who performed the interventions and did not normally work in the ICU.

### Intervention group

The intervention group received the ABCDE bundle. The completed Template for Intervention Description and Replication (TIDieR) [18] provided in Additional file 2 provides a detailed description regarding the intervention for replication in future studies.

In brief, the ABCDE bundle education program commenced 2 months prior to study commencement. Multimodal education was delivered via unit-based presentations and simulation in order to familiarise staff with procedures and to practice emergency responses. Ongoing support was provided during the trial. Nursing, medical and allied health staff who provide exercise interventions in the trial were experienced critical care clinicians.

The ABCDE bundle was a standardised and protocolised complex care bundle that was integrated into daily patient care activities and delivered by the appropriate member of the treating multidisciplinary team at various times each day. The implementation of the bundle was facilitated by using computer software that provided staff care prompts. That is, the protocol components were embedded into the patient record computerised information system (CIS). Each component of the bundle was prescribed by nursing or physiotherapy staff following completion of a safety screen. The CIS provided an alert when bundle components were due for action. A 1-h window was provided so that other ICU activities could be considered. An electronic signature was required to confirm that each component of the bundle had or had not been completed.

The Awakening and Breathing Coordination (ABC) component of the bundle required the completion of a safety screen within the CIS to determine whether it was safe to interrupt sedation and commence a spontaneous awakening trial (SAT). If the SAT was successful, a Spontaneous Breathing Trial (SBT) safety screen was performed prior to testing pressure support ventilation or a T-piece trial. Mechanical ventilation was recommenced if the patient failed the SBT. The intensivist considered extubation if the SBT was passed within 30 min.

The Delirium monitoring and management component ensured all patients received routine pain, sedation and delirium assessment using standardised and validated assessment tools [19, 20]. Pain was assessed every 2 h with the Numeric Rating Scale (NRS) if conscious or the Critical Care Observation Tool (CCOT) if unconscious. Continuous infusions of Fentanyl and/or Remifentanil were titrated to keep NRS less than 4 or CCOT less than 3. Level of alertness was monitored every 4 h with the Richmond Agitation and Sedation Scale (RASS). Sedation was optimised by keeping the RASS between light sedation (− 2) to alert and calm (0). Propofol and/or Dexmedetomidine were recommended for the patients in the intervention group. The CAM-ICU was performed every 12 h (8 am and 8 pm) to determine if the patient was delirious.

The early exercise and mobility component of the bundle required the completion of a screen within the CIS to ensure the patient met safety criteria. If the patient did not pass the safety screen, they received passive range of motion exercise and sitting position three times a day in bed. The nurse or physiotherapist determined the patient’s capacity for independent movement. Patients progressed through four levels of progressive activity, receiving the highest level they could manage*.* The nurse created a nursing order in the CIS which would appear in the CIS at the appropriate time throughout the day. The nurse was provided a 1-h window either side of the prescribed time to allow other patient therapies to occur.

Nursing, medical and allied health staff involved in providing interventions and those involved in performing outcome measurements were experienced critical care clinicians and were deemed competent to perform the rehabilitation strategies. Safety guidelines especially with regard to management of endotracheal tubes and invasive lines during exercise and mobility were provided to all ICU staff involved in patient care. Safety strategies were effective as no adverse events occurred during the trial.

### Control group

The control group received standard medical, nursing and allied health care with routine pain, sedation and delirium assessment using the Numeric Rating Scale (NRS) if conscious or the Critical Care Observation Tool (CCOT) if unconscious, the Richmond Agitation and Sedation Scale, and the Confusion Assessment Method-ICU (CAM-ICU).

Interruption of sedative and opiate infusions was performed at the discretion of the ICU consultant on duty and would generally occur following the morning handover round (8.00 am). The decision to progress to cessation of sedation was based on information regarding the patient’s clinical progress during the previous 12 to 24 h. There were no standardised procedures to guide spontaneous breathing trials. Nursing staff would closely monitor the patient’s neurological, haemodynamic and respiratory status. The intensive care specialist would make the decision whether to progress to extubation or to resedate the patient.

There were no standardised approaches to the provision of exercise and mobility at this site. Nursing staff did not routinely provide patient exercise. Physiotherapists provided physical exercise for each patient on an ad hoc basis. The degree of exercise ranged from no exercise to passive range of motion to sitting out of bed and was delivered at the discretion of individual physiotherapists.

### Primary outcome assessment data

The Functional Independence Measure (FIM) quantified the participant’s functional and cognitive status at both ICU and hospital discharge. This tool is validated for use in the critically ill population [21, 22] and provides reliable information regarding patient functional change during rehabilitation across various hospital settings [23].

### Secondary outcome assessment data

The Physical Function ICU Test-scored (PFIT-s) is a reliable and responsive measure of the physical function and potential patient physical limitations of critically ill patients [24]. This test was developed in Australia [24] and has recently been validated for patient cohorts in the United States (US) requiring mechanical ventilation for 4 days or longer [25]. In our study, the PFIT-s was used in addition to the FIM to provide a specific functional test for ICU patients at ICU discharge.

Physiotherapists who performed the PFIT-s and FIM assessments and Occupational Therapists who performed the FIM assessments were trained and credentialed in the use of the tools. Assessments were timed to be performed within 24 h of both discharge from the ICU using FIM and PFIT-s and discharge from the acute hospital using FIM.

The Short-Form (36) Health Survey (SF-36v2™) provided a baseline and post discharge measure of participants’ health-related quality of life (HRQOL). The SF-36v2™ is a validated and reliable tool [26] and has been used to assess the health status of ICU patients prior to hospitalisation and after discharge [27]. Study data collectors delivered the questionnaires during one-on-one personal interviews with participants or their next of kin when an inpatient of the ICU and by telephone interview at the 90-day post discharge follow-up. Responses provided by next of kin to HRQOL questions within the SF-36 have been validated [28] with a significant correlation shown between responses provided by patients and their next of kin [29].

Whilst the main purpose of the study was to test feasibility, the research team also collected health outcome data including duration of stay in the ICU and hospital, duration of mechanical ventilation, and mortality.

### Trial feasibility - Participant recruitment and retention rates

Enrolment logs were recorded for all patients who met eligibility criteria. Pre-screen failure logs were kept for patients who met the inclusion criteria but were unable to be enrolled. Reasons for pre-screen failure were recorded and data entered into an excel spreadsheet. Recruitment success was defined as 80% of eligible participants agreeing to be enrolled in the study. Successful retention was defined by less than 10% attrition rate for those participants who had survived to the 90-day post discharge assessment.

### Trial feasibility - Intervention fidelity

The bedside nurse recorded each component of the ABCDE bundle as it was delivered within the CIS. The research assistants retrospectively recorded this data on a case report form. Successful adherence to the protocol was defined as the administration of the entire prescribed ABCDE bundle on at least 80% of ventilated days.

Awakening and Breathing Coordination: Participant records were checked for the presence of completed Spontaneous Awakening Trial (SAT) and Spontaneous Breathing Trial (SBT) safety screen, and whether a SAT and SBT was performed if appropriate. Full compliance was considered if a SAT and SBT if appropriate had been administered each day.

Delirium monitoring and management: Assessment of sedation and delirium status using the Richmond Agitation Sedation scale [20] and the CAM-ICU [19] were recorded.

Early Exercise: Protocol adherence was determined when the participant performed an appropriate level of exercise each day.

### Trial feasibility - Barriers to the implementation of the intervention

Reasons for not completing components of the ABCDE bundle were recorded by the bedside nurse. These records were stored at the bedside until collection by the research team at participant discharge from the ICU.

### Trial feasibility - Adverse events

Participants who suffered an adverse event were to be immediately reviewed by the intensivist and the event recorded in detail. Adverse events were to be reviewed by an independent safety monitor at the recruitment of participant 10 and 20 or before if indicated.

### Trial feasibility - Collection of outcome data.

Primary and secondary outcome measures were performed at four time points during the trial. A baseline assessment occurred at enrolment into the study, with further assessments performed within 24 h of ICU discharge, hospital discharge and the final Health-Related Quality of Life (HRQOL) assessment performed at 90 days post discharge from hospital.

### Statistical analysis

The feasibility study was designed to test our procedures and estimate the proportions of our participants who would meet our feasibility objectives in a powered RCT.

Demographic and participant characteristics were summarised by using mean and standard deviation for continuous variables and number and percent for categorical variables. Descriptive statistics were used to report feasibility outcomes. Participant recruitment and retention rates, the adherence to the trial intervention, barriers to the implementation of the intervention, and the feasibility of collecting outcome assessment data were summarised and reported as frequencies and proportions or as free text. Functional and quality of life outcomes were expressed as mean and standard deviation.

Between groups inferential comparisons were not performed as the study was not powered for this analysis. All analyses were based on the intention-to-treat principle using complete case data only and were performed using IBM SPSS software, version 21.0.

## Results

Thirty critically ill patients were randomised. The mean age of the recruited patients was 57.7 years (SD = 13.8). Seventeen (56.7%) of the cohort were female representing a female-to-male ratio of 1.3:1. Average severity of illness score (APACHE II) was 14.3 (SD = 5.4). Patients were admitted to the ICU with a variety of clinical diagnoses. All four patients admitted with gastrointestinal disorders were randomised to the intervention group (26%). This may have skewed results as this group had a lengthy ICU stay (mean 16.2 days, SD 11.6) and increased duration of ventilation (mean 14.1 days, SD 11.3). Mortality data was collected to the 90-day follow-up. However, all mortality occurred prior to hospital discharge. Baseline characteristics of the recruited participants are shown in Table 1.

**Table 1:** Baseline characteristics of participants

Variable	Mean (SD) or *n* (%)	
	Intervention (*N = 15*)	Control (*N = 15*)
Age in years	54.9 (15.9)	60.6 (11.0)
APACHE II score	14.9 (5.9)	13.7 (5.0)
Gender (Female)	6 (40.0%)	11 (73.7%)
ICU length of stay (days)	12.1 (6.9)	10.0 (6.3)
Duration of ventilation (days)	10.1 (7.0)	7.4 (5.2)
Hospital length of stay (days)	16.3 (9.3)	17.5 (13.6)
Mortality	4 (13.3%)	1 (3.3%)
Diagnosis		
• Respiratory	5 (33.3%)	6 (40.0%)
• Sepsis	4 (26.7%)	7 (46.7%)
• Gastro-intestinal	4 (13.3%)	0 (0.0%)

*APACHE* Acute Physiology and Chronic Health Evaluation; *ICU* intensive care unit; *SD* standard deviation

The results of this study are categorised and assessed against 14 methodological items suggested by Shanyinde et al. [30] and Bugge et al. [31] to evaluate a feasibility study. A summary of the items is provided in Table 2.

**Table 2:** Summary of findings against 14 methodological issues for feasibility research

Methodological items	Findings	Evidence
1. What factors influenced eligibility and what proportion of those approached were eligible?	Ineligibility for inclusion was mainly due to not expected to survive, having a neuromuscular illness, or having an advanced health directive or acute care plan.	30 out of 37 eligible patients (81.1%) agreed to participate in the trial.
2. Was recruitment successful?	Yes. Recruiting success was defined as 80% of eligible participants agreeing to participate and were enrolled in the study. Review of inclusion and exclusion criteria will facilitate more timely recruitment.	81.1% of eligible participants agreed to participate and were enrolled in the study.
3. Did eligible participants consent?	Yes. There was high conversion to consent.	30 out of 37 (81.1%) eligible patients or their substitute decision maker consented to participate.
4. Were participants successfully randomised?	Yes. Randomisation procedures worked well.	Table 1 shows variation between the intervention and control group as may occur with small sample size
5. Were blinding procedures adequate?	Yes. Assessors of the FIM and PFIT-s remained blinded to participant assignment throughout the trial.	Participant treatment group information was not provided to assessors. Assessors did not work in ICU.
6. Did participants adhere to the intervention?	Yes. Successful adherence to the Intervention was defined as participants receiving at least 80% of the components of the intervention on each ventilated day.	Awakening and Breathing Coordination: A daily SAT and a SBT was provided on 105 of a total of 131 ventilated days (80.2%). Delirium monitoring and management: RASS score was completed on 100% of ventilated days, CAM–ICU was completed on 97.4% of ventilated days. Early exercise and mobility: The intervention group participated in a total of 432 exercise sessions out of a total of 479 prescribed sessions (90.2%).
7. Was the intervention acceptable to the participants?	Both participants and their families were keen for the patient to receive the intervention. Acceptability was measured by participant refusal to comply with therapy.	1 out of 47 exercise sessions (2.1%) not delivered was related to patient refusal.
8. Was it possible to calculate intervention costs and duration?	An economic evaluation was not conducted as part of this study	
9. Were outcome assessments completed?	Reasons for non-completion of the assessment included mortality, transfer to other facilities, refusal of the assessment at hospital discharge and one patient remained an in-patient at the time of writing this report.	A total of 25 (83.3%) FIMs were performed at ICU discharge and 24 (80%) at hospital discharge. 24 patients (80%) completed the PFIT-s assessment at ICU discharge. 29 (96.7%) HRQOL assessments were completed during the initial ICU admission. 23 (76.7%) assessments were completed at 90 days post discharge.
10. Were outcomes measured those that were the most appropriate outcomes?	All outcomes were deemed appropriate and valid.	The PFIT-s has a small floor and ceiling effect that may exclude functional assessments in some ICU patients [45].
11. Was retention to the study good?	Successful retention in the study was defined by less than 10% attrition rate for those patients who had survived to the 90 days post discharge assessment.	Five (16.7%) of the 30 participants had died within 90 days following hospital discharge. Twenty three (92.0%) of the remaining 25 participants, were successfully followed up. Two participants were lost to follow up due to non-contactability (*n* = 1) and withdrawal of consent (*n* = 1).
12. Were the logistics of running a multi-centre trial assessed?	No. This was not assessed as this is a single-centre trial.	
13. Did all components of the protocol work together?	The components of both the study itself and the complex intervention worked well together.	Study processes were completed and met all pre-determined criteria at the end of the study.
14. Did the feasibility/pilot study allow a sample size calculation for the main trial?	No. A sample size calculation for a future RCT was not calculated.	Our feasibility study did not provide a meaningful effect size estimate for planning a subsequent RCT. This is due to the imprecision inherent in data from small sample sizes [46].

### Eligibility

Between May and December 2015, 433 patients were screened for suitability for inclusion in the trial. A total of 37 patients met the inclusion criteria of which three were not enrolled as they were outside the time for recruitment, one declined consent, one was homeless and could not be followed up, an interpreter was unable to be found for a non-English speaking patient and one patient was related to a research team member. A summary of patient eligibility is illustrated in the CONSORT flow diagram (Fig. 1).

### Recruitment and consent

Recruitment and consent processes performed by dedicated research personnel were deemed successful. Ultimately, 30 eligible patients (81.1%) consented and were randomised either into the intervention (*n* = 15) or control group (*n* = 15).

### Randomisation procedures

Randomisation processes worked smoothly with an unpredictable assignment to comparison groups. However, there was a noted imbalance in demographic data with a disproportion in age and gender assignments between the groups.

### Blinding procedures

The outcome assessors who completed the FIM and PFIT-s remained blinded to group assignment throughout the course of the trial. It was not possible to blind the research team, the treating multidisciplinary team or the patient to group assignment.

### Adherence to the intervention

Adherence to this complex intervention exceeded the defined success rate of 80% of sessions received each mechanically ventilated day.

#### Awakening and breathing coordination

The intervention group received a daily SAT and a SBT if appropriate determined by a positive safety screen on 105 of a total of 131 ventilated days (80.2%). Of these, 45 SBT sessions (34.4%) were ordered by the consultant on duty even if the patient had not passed a safety screen.

#### Delirium monitoring and management

Documentation of the RASS score was completed on 100% of ventilated days, whilst the CAM–ICU was completed on 97.4% of ventilated days. Delirium was noted to be present with a positive CAM–ICU on 39.6% of mechanically ventilated days for the entire cohort of participants.

#### Early exercise and mobility

The intervention group participated in a total of 432 exercise sessions out of a total of 479 prescribed sessions (90.2%).

Safety screens built into the CIS met expectations. There were no adverse events during intervention sessions.

### Acceptability of the intervention–barriers

A total of 479 exercise sessions were prescribed for the intervention participants during the trial. Of these, 37 (7.7%) sessions were not delivered and of those 34 (91.9%) clearly had a reason documented. The reasons that participants failed to receive their exercise session is presented in Table 3.

**Table 3:** Barriers to exercise

Reason/Barrier	Number	Percentage
Haemodynamic/respiratory instability	8	21.6
Sleeping	7	18.9
Procedures	6	16.2
Dialysis	3	8.1
Agitation/delirium	2	5.4
Heavily sedated; complex wound; patient paralysed; patient refusal; pain; prone position; end of life care; invasive devices.	1 each	2.7 each
Missing data	3	8.1

### Outcome assessment

A total of 25 (83.3%) FIM assessments were performed at ICU discharge and 24 (80%) at hospital discharge.

Reasons for non-completion of the assessment included mortality, with a total of three deaths at ICU discharge and a further death at hospital discharge; two participants were transferred to other facilities, one participant refused the assessment at hospital discharge and one participant remained a hospital in-patient at the time of project completion.

Twenty-four participants (80%) completed the PFIT-s assessment at ICU discharge. Reasons for non-completion were mortality (*n* = 3, 10%); participant was transferred to another facility (*n* = 2, 6.7%) and one participant refused to be assessed (*n* = 1, 3.3%) due to fatigue.

HRQOL was assessed with the SF-36 v 2.0 at baseline and again at 90 days post discharge from hospital. Twenty-nine (96.7%) baseline assessments were completed during the initial ICU admission. One participant died during the admission, and it was deemed inappropriate to seek the baseline assessment. At 90 days post discharge, 23 (76.7%) follow-up assessments were complete. A total of 5 (16.7%) participants died during the trial period related to their clinical condition, whilst a further 2 (6.7%) refused to participate. Despite longer length of stay and duration of ventilation, the intervention group mean scores indicated better functional and quality of life results at discharge. However, the sample size was too small to provide reliable interpretation.

The entire participant group received RASS assessment on 227 (100%) ventilated days. CAM-ICU assessments were performed on 221 (97.4%) ventilated days. The management component of delirium was not formally recorded, as interventions had been suggested but not prescribed. The primary and secondary outcome assessment data is summarised in Table 4.

**Table 4:** Summary of outcome data for study groups

Outcome measure	All participants Mean (SD) (*N = 30*)	Intervention group Mean (SD) (*N = 15*)	Control group Mean (SD) (*N = 15*)
FIM at ICU discharge	45.5 (18.4)	46.7 (16.8)	44.6 (20.2)
FIM at hospital discharge	95.4 (26.1)	95.8 (29.6)	95.2 (24.7)
PFIT-s at ICU Discharge	6.5 (1.2)	7.0 (1.1)	6.15 (1.2)
SF-36 PCS at baseline	37.5 (11.9)	32.7 (11.1)	41.6 (11.4)
SF-36 PCS at 90 day follow-up	40.6 (11.4)	43.8 (12.0)	37.9 (10.7)
SF-36 MCS at baseline	43.3 (14.1)	45.0 (14.4)	41.8 (14.1)
SF-36 MCS at 90 day follow-up	43.4 (16.0)	47.4(16.0)	40.3 (15.9)

### Selection of most appropriate outcome test

The outcome assessment tests provided robust clinimetric measurements of the participant’s physical, functional and HRQOL.

### Trial feasibility—retention

Five (16.7%) of the 30 participants had died within 90 days post hospital discharge. Twenty-three (92.0%) of the remaining 25 participants, were successfully followed up. Two participants were lost to follow-up due to non-contactability (*n* = 1) and withdrawal of consent (*n* = 1).

### Synergy of the components of the trial

The study protocol was robust evidenced by successful implementation of a new complex intervention at the study site which met study objectives. This suggests that the study protocol requires very minor adjustment to provide evaluation of the ABCDE bundle in an appropriately powered trial.

## Discussion

There are few studies that explore ‘whole body’ rehabilitation in the ICU. In the literature, the term ‘rehabilitation’ frequently refers to mobility or exercise programs exclusively [22, 32–36]. However, rehabilitation strategies in the ICU should aim to restore physical, functional and cognitive capabilities [37]. This may potentially be achieved using a bundled approach to treat and prevent both ICU-acquired weakness and delirium.

Health care interventions require rigorous evaluation, and most health researchers agree that this is best achieved with an RCT. However, complex interventions made up of various components like the ABCDE bundle pose additional methodological challenges. The UK MRC guidance on designing and evaluating complex interventions [14] explicitly recommend thorough feasibility work as an important initial step in the phased approach to identifying problems that might occur in an RCT of a complex intervention. This important preparatory stage aims to deliver an RCT that is both robust (internal validity) and generalizable in a real world context (external validity) [31]. Important methodological criteria have been assessed within this study to determine whether a definitive RCT is feasible.

Target enrolment of an adequate number of participants to test the study protocol was achieved. However, recruitment of participants was accomplished in 8 months. We are mindful that an adjustment to the recruitment strategy is required to ensure that recruitment goals meet timely project completion.

The selection of participants who required a minimum of 72 h of invasive ventilation resulted in a cohort with a prolonged stay in the ICU. As it was not the intent of the study to focus only on long stay patients, and as little as 1 day of bed rest has been shown to have an impact on muscle weakness [38], reducing the time for inclusion would provide relevance to the wider ICU population.

The exclusion criteria relied on subjective assessments only. Examination of functionality using a validated tool would have strengthened objective assessment. Although exclusion criteria were designed to avoid the confounding effects of pre-existing cognitive and medical conditions, eligibility numbers were lower than expected indicating the criteria was restrictive. Potential cohorts of the normal ICU population were omitted from the study which would negatively impact on the generalisability of the results. For instance, patients with neuromuscular disorders were excluded from the study, yet are capable of performing some level of exercise and mobility and importantly often require rehabilitation post ICU discharge [39]. Revision of the exclusion criteria to also include patients suffering premorbid functional impairment will strengthen the relevance of a larger RCT.

Randomisation processes worked efficiently and facilitated unpredictable assignment to the control and the intervention group. Participants were randomised once they had been mechanically ventilated for 48 h and were expected to remain ventilated for at least 72 h. Although limited, previous research confirms early rehabilitation interventions that incorporate both prevention of delirium and early physical exercise can optimise the short term outcomes and long term quality of life for intensive care unit survivors [15]. In this study, patients had frequently been administered potentially delirium inducing sedation prior to randomisation negating the positive effects of the ABCDE bundle. Revision of recruitment processes to ensure recruitment at 24 h following the commencement of mechanical ventilation would facilitate earlier implementation of interventions.

Retention throughout the trial to the 90-day follow-up was deemed successful with only two of the surviving participants lost to follow-up. Participants’ responses to follow-up phone calls were very positive with the researcher often providing reassurance regarding physical, emotional and psychological recovery. Research personnel were responsive and friendly, characteristics which have been shown to foster participant motivation and retention in a previous study [40].

We have demonstrated that the ABCDE bundle can be reliably implemented in an Australian ICU with good intervention fidelity. Awakening and breathing coordination protocols focussed on safe cessation from sedative drugs and timely preparation for extubation. Medical staff incorporated nurse initiated safety screens with clinical judgement although SBTs were frequently ordered by the medical staff regardless of whether the patient had passed the safety screen.

Long-term patients that experienced difficulty weaning from the ventilator presented a clinical challenge. However, we were mindful that the inclusion of delirium management and early exercise would be particularly beneficial for this cohort of patient. Trial participants received a positive CAM-ICU assessment on 39.6% of mechanically ventilated days. This is a similar rate to the before/after US study which found a significant reduction in prevalence and duration of delirium following the introduction of the ABCDE bundle [41]. Although the ICU team improved delirium assessment, prevention and management remained an issue. A scripted pharmacological and non-pharmacological regime would be key to the future success of an RCT.

Staff were encouraged to provide a minimum of passive range of motion exercises to all participants in the intervention group. A 2-h window around the prescribed exercise session permitted necessary flexibility for clinical procedures. The standard bed in our ICU facilitated a ‘sit in full chair position’ without removing the patient from the bed. Generalisation of our protocol to other sites would be impacted by the lack of this or similar equipment. Prescription of exercise as an actionable task in the CIS promoted protocol adherence.

Potential contamination of the care of the control group was considered. The intervention group received the standardised and protocolised ABCDE bundle via a prescription within the clinical information system which once ordered could not be ignored or deleted resulting in the delivery of consistent and standard care every day. To provide additional control in our pilot study, only the intervention group had access to the ABCDE protocols via the clinical information system. The protocols were not available in any other format. Alternatively, the control group received care dependent on the medical officers, physiotherapists, occupational therapist, nursing decisions made daily with no use of protocol. Variation in the control group occurred related to different clinical practice and effort of individuals.

A future RCT would benefit from the inclusion of data related to contamination of the control group.

Family engagement and involvement was critical to the success of this study. Healthcare professionals worked in partnership with families to promote participant support and encouragement. Family members viewed the completion of increasingly difficult levels of exercise as a positive affirmation that their relative was recovering. Improving family satisfaction by encouraging participation and communication has been shown to decrease psychological distress and is considered a critical component of the provision of quality care in ICU [42]. Importantly, latest literature includes an “F” for family engagement in the ABCDE bundle acknowledging the value of this relationship in the recovery process [43].Thus, an ABCDEF bundle will be implemented in the future RCT.

Our study has some limitations. It is possible that some clinicians would have provided the intervention to all patients. A future RCT would benefit from the inclusion of data related to potential contamination of the control group. It is important to emphasise that our feasibility study was not designed or appropriately powered to test a hypothesis that objective will be achieved in a future RCT. A review of literature supports the assertion that hypothesis testing in a feasibility study is inappropriate [30, 44, 45]. Therefore, we cannot draw conclusions regarding the ABCDE’s bundle effect on functional or HRQOL outcomes. Enrolment at a single site limits the generalisability of our results, and bias may have been introduced related to our inability to blind participants and ICU staff.

## Conclusion

Functional disability and reduced quality of life remains problematic for ICU survivors highlighting the requirement for further investigation to guide quality care improvements in our ICUs. This feasibility study was designed to test important aspects of our research methodology to ensure a future appropriately powered RCT would be robust. With only minor adjustment, we can assert that a large-scale RCT to test the effect of the ABCDEF bundle’s impact on critically ill survivors functional and quality of life indicators will be feasible.

## Additional files

Additional file 1: CONSORT checklist of information to include when reporting a pilot trial. (DOCX 17 kb)

Additional file 2: Template for Intervention Description and Replication (TIDieR) checklist. (DOCX 3357 kb)

## Abbreviations

ABC: Awakening and Breathing Coordination
ABCDE: Awakening and Breathing Coordination, Delirium monitoring and management, and Early exercise and mobility
ABCDEF: Awakening and Breathing Coordination, Delirium monitoring and management, and Early exercise and mobility; Family engagement
CAM–ICU: Confusion Assessment Method–ICU
CCOT: Critical Care Observation Tool
CIS: Computer Information System
FIM: Functional Independence Measure
HRQOL: Health-related quality of life
ICU: Intensive Care Unit
MRC: Medical Research Council
NRS: Numeric Rating Scale
PAD: Pain, Agitation, and Delirium
PFIT-s: Physical Function ICU Test- scored
RASS: Richmond Agitation Scale
RCT: Randomised controlled trial
SAT: Spontaneous Awakening Trial
SBT: Spontaneous Breathing Trial
SF-36: Short Form – 36

## References

[1] Reade MC, Finfer S (2014). Sedation and delirium in the intensive care unit. N Engl J Med.

[2] Schweickert WD, Pohlman MC, Pohlman AS, Nigos C, Pawlik AJ, Esbrook CL, Spears L, Miller M, Franczyk M, Deprizio D (2009). Early physical and occupational therapy in mechanically ventilated, critically ill patients: a randomised controlled trial. Lancet.

[3] Herridge MS, Tansey CM, Matté A, Tomlinson G, Diaz-Granados N, Cooper A, Guest CB, Mazer CD, Mehta S, Stewart TE (2011). Functional disability 5 years after acute respiratory distress syndrome. N Engl J Med.

[4] Barr J, Fraser GL, Puntillo K, Ely EW, Gelinas C, Dasta JF, Davidson JE, Devlin JW, Kress JP, Joffe AM (2013). Clinical practice guidelines for the management of pain, agitation, and delirium in adult patients in the intensive care unit. Crit Care Med.

[5] Trogrlic Z, van der Jagt M, Bakker J, Balas MC, Ely EW, van der Voort PH, Ista E (2015). A systematic review of implementation strategies for assessment, prevention, and management of ICU delirium and their effect on clinical outcomes. Crit Care.

[6] Brummel NE, Jackson JC, Pandharipande PP, Thompson JL, Shintani AK, Dittus RS, Gill TM, Bernard GR, Ely EW, Girard TD (2014). Delirium in the intensive care unit and subsequent long-term disability among survivors of mechanical ventilation. Crit Care Med.

[7] Balas MC, Vasilevskis EE, Burke WJ, Boehm L, Pun BT, Olsen KM, Peitz GJ, Ely EW (2012). Critical care nurses’ role in implementing the “ABCDE bundle” into practice. Crit Care Nurse.

[8] Harper S (2015). Implementation plan for the ABCDE bundle.

[9] Barnes-Daly A, Phillips M, Ely G, Wesley E: ICU liberation: Using the ABCDEF bundle to improve outcomes in 7 California community ICUs In: Critical Care Congress. vol. Volume 43 Orlando, Florida: Critical Care Medicine:; 2015: 11.

[10] Balas MC, Vasilevskis EE, Olsen KM, Schmid KK, Shostrom V, Cohen MZ, Peitz G, Gannon DE, Sisson J, Sullivan J (2014). Effectiveness and safety of the awakening and breathing coordination, delirium monitoring/management, and early exercise/mobility bundle*. Crit Care Med.

[11] Eldridge SM, Chan CL, Campbell MJ, Bond CM, Hopewell S, Thabane L, Lancaster GA (2016). CONSORT 2010 statement: extension to randomised pilot and feasibility trials. Pilot Feasibility Stud.

[12] Zealand. CoICMoAaN: Minimum standards for intensive care units. 2011, IC-01.

[13] Levati S, Campbell P, Frost R, Dougall N, Wells M, Donaldson C, Hagen S (2016). Optimisation of complex health interventions prior to a randomised controlled trial: a scoping review of strategies used. Pilot Feasibility Studies.

[14] Craig P, Dieppe P, Macintyre S, Michie S, Nazareth I, Petticrew M. Developing and evaluating complex interventions: the new Medical Research Council guidance. BMJ. 2008;337:a1655. 10.1136/bmj.a1655 PMC2769032 18824488

[15] Sosnowski K, Lin F, Mitchell ML, White H (2015). Early rehabilitation in the intensive care unit: an integrative literature review. Aust Crit Care.

[16] Dobkin B (2009). Progressive staging of pilot studies to improve phase III trials for motor interventions. Neurorehab Neural Repair.

[17] Plow M, Moore S, Kirwan J, Frost F, Katzan I, Jaeger S, Alberts J (2013). Randomized controlled pilot study of a SystemCHANGETM weight management intervention in stroke survivors: rationale and protocol. Trials.

[18] Hoffmann TC, Glasziou PP, Boutron I, Milne R, Perera R, Moher D, Altman DG, Barbour V, Macdonald H, Johnston M, et al. Better reporting of interventions: template for intervention description and replication (TIDieR) checklist and guide. BMJ. 2014;348:g1687. 10.1136/bmj.g1687 24609605

[19] Ely EW, Margolin R, Francis J, May L, Truman B, Dittus R, Speroff T, Gautam S, Bernard GR, Inouye SK (2001). Evaluation of delirium in critically ill patients: validation of the Confusion Assessment Method for the Intensive Care Unit (CAM-ICU). Crit Care Med.

[20] Ely EW, Truman B, Shintani A, Thomason JW, Wheeler AP, Gordon S, Francis J, Speroff T, Gautam S, Margolin R (2003). Monitoring sedation status over time in ICU patients: reliability and validity of the Richmond Agitation-Sedation Scale (RASS). JAMA.

[21] Schweickert WD, Gehlbach BK, Pohlman AS, Hall JB, Kress JP (2004). Daily interruption of sedative infusions and complications of critical illness in mechanically ventilated patients. Crit Care Med.

[22] Zanni JM, Korupolu R, Fan E, Pradhan P, Janjua K, Palmer JB, Brower RG, Needham DM (2010). Rehabilitation therapy and outcomes in acute respiratory failure: an observational pilot project. J Crit Care.

[23] Ottenbacher KJ, Hsu Y, Granger CV, Fiedler RC (1996). The reliability of the functional independence measure: a quantitative review. Arch Phys Med Rehabil.

[24] Denehy L, de Morton NA, Skinner EH, Edbrooke L, Haines K, Warrillow S, Berney S (2013). A physical function test for use in the intensive care unit: validity, responsiveness, and predictive utility of the physical function ICU test (scored). Phys Ther.

[25] Nordon-Craft A, Schenkman M, Edbrooke L, Malone DJ, Moss M, Denehy L (2014). The physical function intensive care test: implementation in survivors of critical illness. Phys Ther.

[26] Brazier JE, Harper R, Jones NM, O'Cathain A, Thomas KJ, Usherwood T, Westlake L (1992). Validating the SF-36 health survey questionnaire: new outcome measure for primary care. BMJ.

[27] Hofhuis JG, Spronk PE, van Stel HF, Schrijvers GJ, Rommes JH, Bakker J (2008). The impact of critical illness on perceived health-related quality of life during ICU treatment, hospital stay, and after hospital discharge: a long-term follow-up study. Chest.

[28] Hofhuis JG, Hautvast JL, Schrijvers AJ, Bakker J (2003). Quality of life on admission to the intensive care: can we query the relatives?. Intensive Care Med.

[29] Hofhuis JG, van Stel HF, Schrijvers AJ, Rommes JH, Spronk PE (2015). ICU survivors show no decline in health-related quality of life after 5 years. Intensive Care Med.

[30] Shanyinde M, Pickering RM, Weatherall M (2011). Questions asked and answered in pilot and feasibility randomized controlled trials. BMC Med Res Methodol.

[31] Bugge C, Williams B, Hagen S, Logan J, Glazener C, Pringle S, Sinclair L (2013). A process for Decision-making after Pilot and feasibility Trials (ADePT): development following a feasibility study of a complex intervention for pelvic organ prolapse. Trials.

[32] Bailey P, Thomsen GE, Spuhler VJ, Blair R, Jewkes J, Bezdjian L, Veale K, Rodriquez L, Hopkins RO (2007). Early activity is feasible and safe in respiratory failure patients. Crit Care Med.

[33] Berney S, Haines K, Skinner EH, Denehy L (2012). Safety and feasibility of an exercise prescription approach to rehabilitation across the continuum of care for survivors of critical illness. Phys Ther.

[34] Burtin C, Clerckx B, Robbeets C, Ferdinande P, Langer D, Troosters T, Hermans G, Decramer M, Gosselink R (2009). Early exercise in critically ill patients enhances short-term functional recovery. Crit Care Med.

[35] Chen YH, Lin HL, Hsiao HF, Chou LT, Kao KC, Huang CC, Tsai YH (2012). Effects of exercise training on pulmonary mechanics and functional status in patients with prolonged mechanical ventilation. Respir Care.

[36] Davis J, Crawford K, Wierman H, Osgood W, Cavanaugh J, Smith KA, Mette S, Orff S (2013). Mobilization of ventilated older adults. J Geriatr Phys Ther.

[37] Parker A, Sricharoenchai T, Needham DM (2013). Early rehabilitation in the intensive care unit: preventing physical and mental health impairments. Curr Phys Med Rehabil Rep.

[38] Fan E, Dowdy DW, Colantuoni E, Mendez-Tellez PA, Sevransky JE, Shanholtz C, Dennison Himmelfarb CR, Desai SV, Ciesla N, Herridge MS (2014). Physical complications in acute lung injury survivors: a two-year longitudinal prospective study. Crit Care Med.

[39] Abresch RT, Han JJ, Carter GT (2009). Rehabilitation management of neuromuscular disease: the role of exercise training. J Clin Neuromuscul Dis.

[40] Dias L, Schoenfeld E, Thomas J, Baldwin C, McLeod J, Smith J, Owens R, Hyman L (2005). Reasons for high retention in pediatric clinical trials: comparison of participant and staff responses in the Correction of Myopia Evaluation Trial. Clin Trials.

[41] Bounds M, Kram S, Speroni KG, Brice K, Luschinski MA, Harte S, Daniel MG (2016). Effect of ABCDE bundle implementation on prevalence of delirium in intensive care unit patients. Am J Crit Care.

[42] Truog RD, Campbell ML, Curtis JR, Haas CE, Luce JM, Rubenfeld GD, Rushton CH, Kaufman DC (2008). Recommendations for end-of-life care in the intensive care unit: a consensus statement by the American College [corrected] of Critical Care Medicine. Crit Care Med.

[43] Balas MC, Barnes-Daly A, Byrum D, Pun BT, Ely W: Helping critically ill patients and families thrive through an ABCDEF approach. In: Critical Connections. vol. August; 2015.

[44] Arain M, Campbell MJ, Cooper CL, Lancaster GA (2010). What is a pilot or feasibility study? A review of current practice and editorial policy. BMC Med Res Methodol.

[45] Parry SM, Denehy L, Beach LJ, Berney S, Williamson HC, Granger CL. Functional Outcomes in ICU - What should we be using? An observational study. Crit Care. 2015;19:127. 10.1186/s13054-015-0829-5 PMC4404223 25888469

[46] Leon AC, Davis LL, Kraemer HC (2011). The role and interpretation of pilot studies in clinical research. J Psychiatr Res.

