# Supplementary material for: A feasibility study of a randomised controlled trial to examine the impact of the ABCDE bundle on quality of life in ICU survivors
Source: Pilot Feasibility Stud. 2018 Jan 11;4:32. doi: 10.1186/s40814-017-0224-x (PMC5765639; doi:10.1186/s40814-017-0224-x)
Supplement: Supplementary file 1 — CONSORT checklist of information to include when reporting a pilot trial. (DOCX 17 kb) [file 40814_2017_224_MOESM1_ESM.docx]

| **Additional file 1. CONSORT checklist of information to include when reporting a pilot trial** | | | |
| --- | --- | --- | --- |
| **Section/Topic** | **Item No** | **Checklist item** | **Reported on page No** |
| **Abstract** | | | |
|  | 1a | Identification as a pilot or feasibility randomised trial in the title | Page 1 |
|  | 1b | Structured summary of pilot trial design, methods, results, and conclusions | Page 3 - 4 |
| **Introduction** | | | |
| Background and objectives | 2a | Scientific background and explanation of rationale for future definitive trial, and reasons for randomised pilot trial | Page 5 - 6 |
|  | 2b | Specific objectives or research questions for pilot trial | Page 6 - 7 |
| **Methods** | | | |
| Trial design | 3a | Description of pilot trial design (such as parallel, factorial,) including allocation ratio. | Page 7 |
|  | 3b | Important changes to methods after pilot trial commencement (such as eligibility criteria) with reasons. | Not Applicable |
| Participants | 4a | Eligibility criteria for participants | Page 7-8 |
|  | 4b | Settings and locations where the data were collected | Page 7 |
|  | 4c | How participants were identified and consented | Page 7 - 8 |
| Interventions | 5 | The interventions for each group with sufficient details to allow replication, including how and when they were actually administered. | Page 8 - 10 and Additional file 2 |
| Outcomes | 6a | Completely defined prespecified assessments or measurements to address each pilot trial objective specified in 2b, including how and when they were assessed. | Page 10 - 13 |
|  | 6b | Any changes to pilot trial assessments or measurements after the pilot trial commenced with reasons | Not applicable |
|  | 6c | If applicable, prespecified criteria used to judge whether or how to proceed with future definitive trial | Not applicable |
| Sample size | 7a | Rationale for numbers in the pilot trial | Page 8 |
|  | 7b | When applicable, explanation of any interim analyses and stopping guidelines | Not applicable |
| Randomisation:  Sequence generation | 8a | Method used to generate the random allocation sequence | Page 8 |
|  | 8b | Type of randomisation; details of any restriction (such as blocking and block size) | Page 8 |
| Allocation concealment mechanism | 9 | Mechanism used to implement the random allocation sequence (such as sequentially numbered containers), describing any steps taken to conceal the sequence until interventions were assigned. | Page 8 |
| Implementation | 10 | Who generated the random allocation sequence, enrolled participants, and assigned participants to interventions. | Page 8 |
| Blinding | 11a | If done, who was blinded after assignment to interventions (e.g. participants, care providers, those assessing outcomes) and how. | Page 8 |
|  | 11b | If relevant, description of the similarity of interventions | Not applicable |
| Analytical methods | 12a | Methods used to address each pilot trial objective whether qualitative or quantitative | Page 10 - 13 |
| **Results** | | | |
| Participant flow (a diagram is strongly recommended) | 13a | For each group, the numbers of participants who were approached and/or assessed for eligibility, randomly assigned, received intended treatment, were assessed for each objective | Page 7-8 and Figure 1 |
|  | 13b | For each group, losses and exclusions after randomisation, together with reasons | Page 14 and Figure 1 |
| Recruitment | 14a | Dates defining the periods of recruitment and follow-up | Page 7. |
|  | 14b | Why the pilot trial ended or was stopped | Not applicable |
| Baseline data | 15 | A table showing baseline demographic and clinical characteristics for each group | Table 1 |
| Numbers analysed | 16 | For each objective, number of participants (denominator) included in each analysis. If relevant, these numbers should be by randomised group. | Page 14 - 17 |
| Outcomes and estimation | 17a | For each objective, results including expressions of uncertainty (such as 95% confidence intervals) for any estimates. If relevant, these results should be by randomised group. | Page 14 – 17 and Table 2 |
| Ancillary analyses | 18 | Results of any other analyses performed that could be used to inform the future definitive trial. | Not applicable |
| Harms | 19 | All important harms or unintended effects in each group | Page 11 |
|  | 19a | If relevant, other important unintended consequences. | Not Applicable |
| **Discussion** | | | |
| Limitations | 20 | Pilot trial limitations, addressing sources of potential bias and remaining uncertainty about feasibility | Page 20 - 21 |
| Generalisability | 21 | Generalisability (applicability) of pilot trial methods and findings to future definitive trial and other studies. | Page 21 |
| Interpretation | 22 | Interpretation consistent with pilot trial objectives and findings, balancing potential benefits and harm, and considering the relevant evidence. | Page 17 - 21 |
|  | 22 a | Implications for progression from pilot to future definitive trial, including any approved amendments. | Page 17 - 21 |
| **Other information** | | |  |
| Registration | 23 | Registration number for pilot trial and name of trial registry | Page 4 |
| Protocol | 24 | Where the pilot trial protocol can be assessed, if available | Page 7 |
| Funding | 25 | Sources of funding and other support (such as supply of drugs), role of funders | Page 23 |
|  | 26 | Ethical approval by research review committee, confirmed with reference number | Page 22 |
