# Supplementary material for: A feasibility study of a randomised controlled trial to examine the impact of the ABCDE bundle on quality of life in ICU survivors
Source: Pilot Feasibility Stud. 2018 Jan 11;4:32. doi: 10.1186/s40814-017-0224-x (PMC5765639; doi:10.1186/s40814-017-0224-x)
Supplement: Supplementary file 2 — Template for Intervention Description and Replication (TIDieR) checklist. (DOCX 3357 kb) [file 40814_2017_224_MOESM2_ESM.docx]

Intervention description using the template for intervention description and replication (TiDieR) checklist

**Item 1. Brief name: Provide the name or a phrase that describes the intervention**

The **ABCDE** bundle of cares - **A**wakening and **B**reathing **C**oordination, **D**elirium monitoring and management, **E**arly exercise and mobility

**Item 2. Why: Describe any rationale, theory, or goal of the elements essential to the intervention**

Evidence reveals that a mechanically ventilated critically ill patient is at significant risk of developing delirium and weakness which can have long term functional and cognitive consequences (Sosnowski et al., 2015). The ABCDE bundle incorporates the best available evidence related to the prevention and management of delirium, preventing the adverse effects related to immobility, sedation and analgesia practices, and ventilator management in the intensive care unit with the ultimate aim of preventing adverse patient outcomes (Balas et al., 2013).

**Item 3. What (materials): Describe any physical or informational materials used in the intervention, including those provided to participants or used in intervention delivery or in training of intervention providers.**

The ABCDE bundle education program was delivered to ICU multidisciplinary staff commencing 2 months prior to study commencement. Multimodal education was delivered via unit based presentations and simulation to assimilate and integrate new knowledge and skill. Information was conveyed at ward meetings and in regular newsletters. Staff were provided opportunities to vent concerns and frustrations prior to the commencement of the study. A survey was provided to the staff to elicit attitude to the bundle and the outcomes we were hoping to achieve. This data was used to alter our education and support strategies.

**Item 4. What (procedures): Describe each of the procedures, activities, and/or processes used in the intervention, including any enabling or support activities.**

The ABCDE bundle was standardised and protocolised by using software to facilitate the clinical application of this quite complex bundle. Our ICU utilises the iMDsoft MetaVision Clinical Information System (CIS). We embedded the protocol components into the system which enabled nursing staff to order the appropriate script for their patient each day. The nurse was alerted by the CIS when components were due for action. A one hour window was provided so that other ICU activities could be taken into account. Intensive training was provided during our education period and ongoing support was provided during the trial.

**Item 5. Who provided: For each category of intervention provider (for example, psychologist, nursing assistant), describe their expertise, background and any specific training given**

The ABCDE bundle team championed and supported the implementation of the bundle in the ICU, provided education and ongoing staff support. This team was made up of the Nurse Unit Manager (post graduate qualified at master’s level, experienced in project and change management), Clinical Nurses (post graduate level qualifications), Nurse Educator (masters levels qualifications), Clinical Facilitator (post graduate level qualifications), and the ICU physiotherapist (bachelor degree qualified). Together, the team developed the education program that was delivered to the ICU staff (see item 3).

On a daily basis, the ABCDE bundle of cares was provided to patients by ICU Registered Nurses (bachelor degree qualified at a minimum, many with post graduate qualification in critical care nursing); allied health team including physiotherapists and occupational therapists (bachelor degree qualified), and medical officers (registrar and specialist level).

**Item 6. How: Describe the modes of delivery (such as face to face or by some other mechanism, such as internet or telephone) of the intervention and whether it was provided individually or in a group**

The ABCDE bundle was integrated into daily patient care activities and components of the bundle were delivered by the appropriate member of the treating multidisciplinary team at various times each day according to each individual participant’s script. The protocol components were embedded into the clinical information system which enabled nursing staff or the physiotherapist to order the appropriate script for the patient each day. The nurse was alerted by the CIS when components were due for action.

**Item 7. Where: Describe the type(s) of location(s) where the intervention occurred, including any necessary infrastructure or relevant features**

Logan Hospital is a 344 bed metropolitan hospital situated on the outskirts of Brisbane in QLD, Australia. All interventions occurred in the Intensive Care Unit. The ICU consists of 8 beds and is classed as a level 2 ICU (CICM., 2011).

**Item 8. When and how much: Describe the number of times the intervention was delivered and over what period of time including the number of sessions, their schedule, and their duration, intensity or dose**

1. **A**wakening and **B**reathing **C**oordination: Spontaneous Awakening Trial (SAT) and Spontaneous Breathing Trial (SBT)

At 07.30 am each day, the RN completed a SAT safety screen (see Figure 1) within the CIS to determine whether it was safe to interrupt sedation received by the intervention group patients. The information was provided to the Consultant at the morning handover round (08.00 am) who then made the decision whether to progress to a SAT (see Figure 2). If the SAT was successful, a SBT safety screen within the CIS was performed to ensure a breathing trial was safe (see Figure 3). If the patient fulfilled safety requirements, a trial pressure support ventilation or a T-piece trial was performed. Criteria to pass or fail the SBT were checked in the CIS. Sedation and mechanical ventilation were recommenced if the patient failed the SBT. The consultant was notified if the SBT had passed within 30 minutes and gave final consideration whether to progress to extubation. A free text area within the CIS provided detail if the consultant decided not to progress to extubation

Figure 1 SAT safety screen


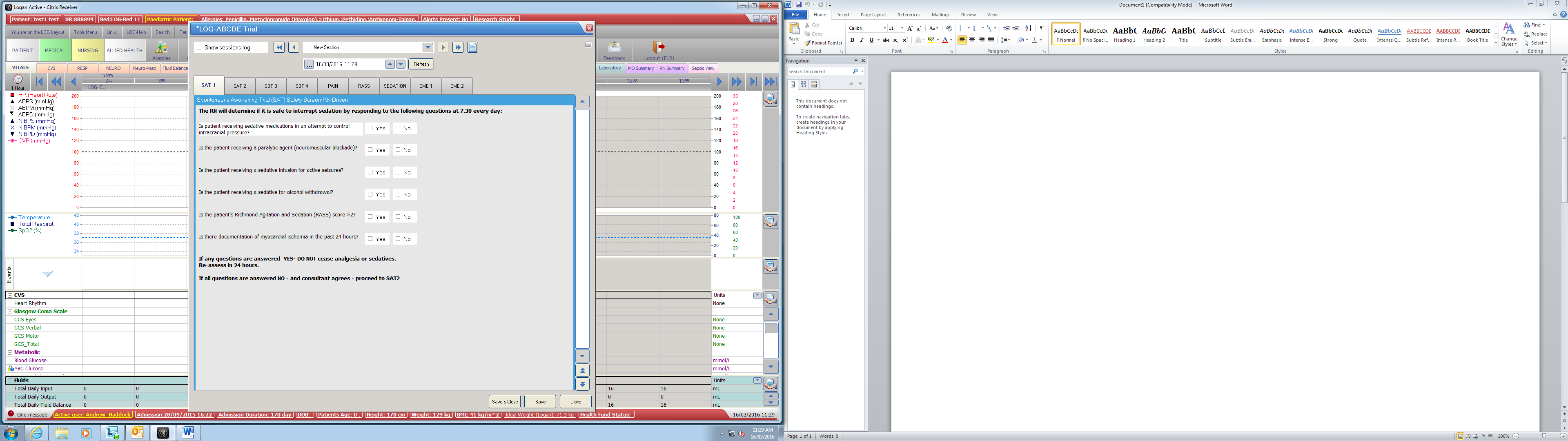


Figure 2 Spontaneous Awakening Trial


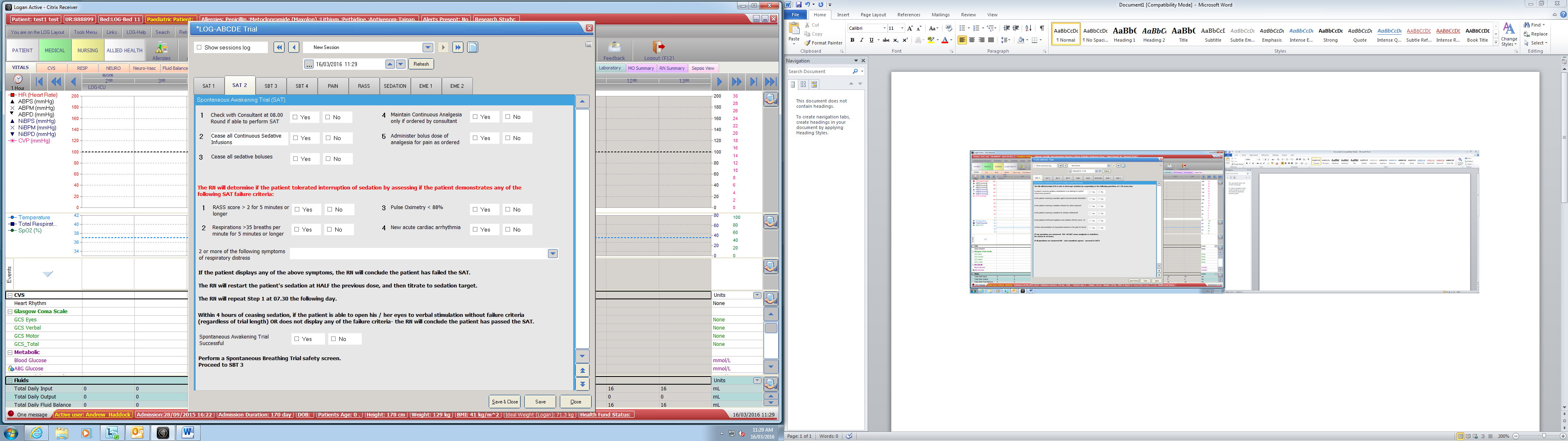


Figure 3 Spontaneous breathing trial safety screen


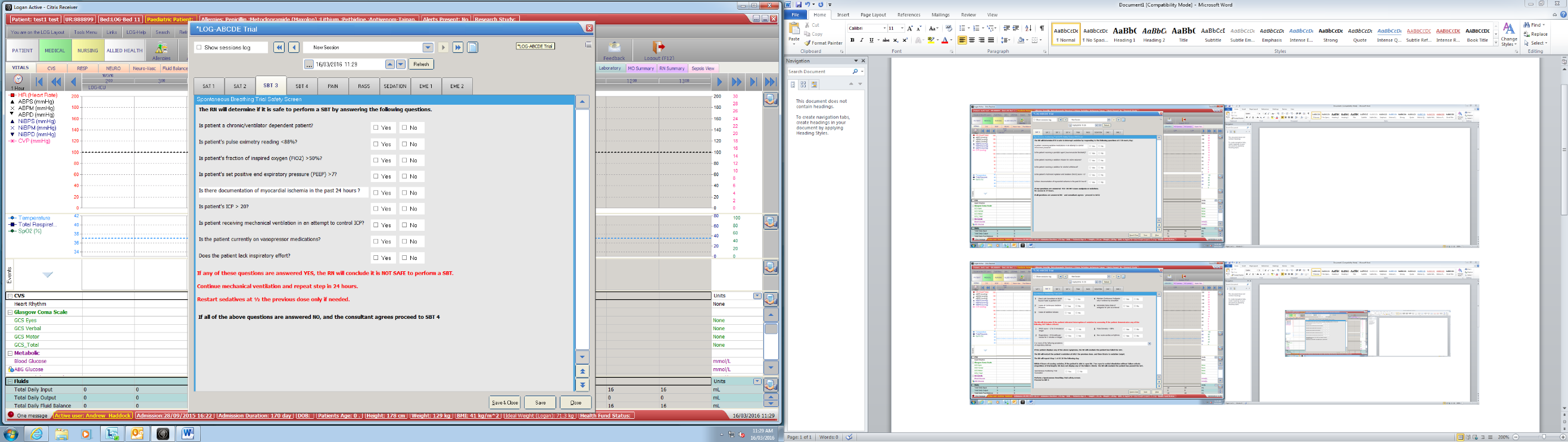


2. **D**elirium monitoring and management

All mechanically ventilated patients received routine pain, sedation and delirium assessment using standardised and validated assessment tools (Ely, Margolin, et al., 2001; Ely et al., 2003). Pain was assessed every 2 hours with the Numeric Rating Scale (NRS) if conscious or the Critical Care Observation Tool (CCOT) if unconscious. Continuous infusions of Fentanyl and/or Remifentanil were titrated to keep NRS less than 4 or CCOT less than 3. Level of alertness was monitored every 4 hours with the Richmond Agitation and Sedation Scale (RASS). Sedation was optimised by keeping the RASS between light sedation (-2) to alert and calm (0). Propofol and/or Dexmedetomidine were recommended for the patients in the intervention group. The CAM-ICU was performed every 12 hours (8am and 8pm) to determine if the patient was delirious. Risk factors were either eliminated or minimised and a therapeutic environment provided for all patients in the intervention group.

3. **E**arly exercise and mobility

Patients in the intervention group were assessed at 07.30 am by the Registered Nurse allocated to the patient to ensure that they met minimum safety criteria. This was scripted as a checklist within the early mobility and exercise safety screen within the CIS (see Figure 4). If a patient did not pass the safety screen, they received level 1 exercise which involved passive range of motion three times a day (06.00, 13.00, 20.00) and sitting position three times a day (06.00, 13.00, 20.00) in the Hill-Rom TotalCare SpO2RT^®^ 2 Therapy Bed – the standard bed in the ICU. Patients who passed the safety screen progressed to the Early Mobility and Exercise tab in the CIS. The Registered Nurse determined the patient’s capacity for independent movement to decide which level of exercise they could receive. The ICU physiotherapist provided guidance if required. Patients progressed through a four level process, receiving the highest level of physical activity they could manage*.* A patient that could lift their arm to command would receive level 2 exercise, whilst a patient that could lift their leg to command would receive level 3 exercises. A patient who had successfully managed level 3 exercises the day previous and could move their arm and leg to command received level 4 exercises which included ambulation. Once the appropriate level of exercise was chosen (see Figure 5), the nurse created a nursing order which acted as a prescription. The prescribed exercise would appear in the CIS at the appropriate time throughout the day. The nurse was provided a one hour window either side of the prescribed time to allow other patient therapies to occur.

Figure 4 Early mobility and exercise safety screen.


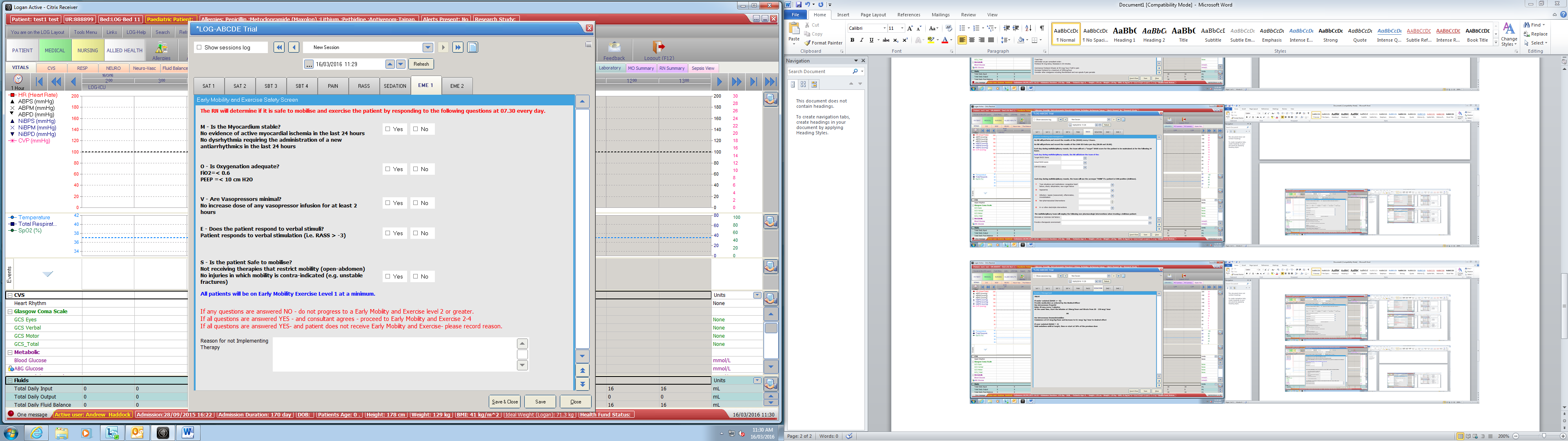


Figure 5 Early mobility and exercise regimen


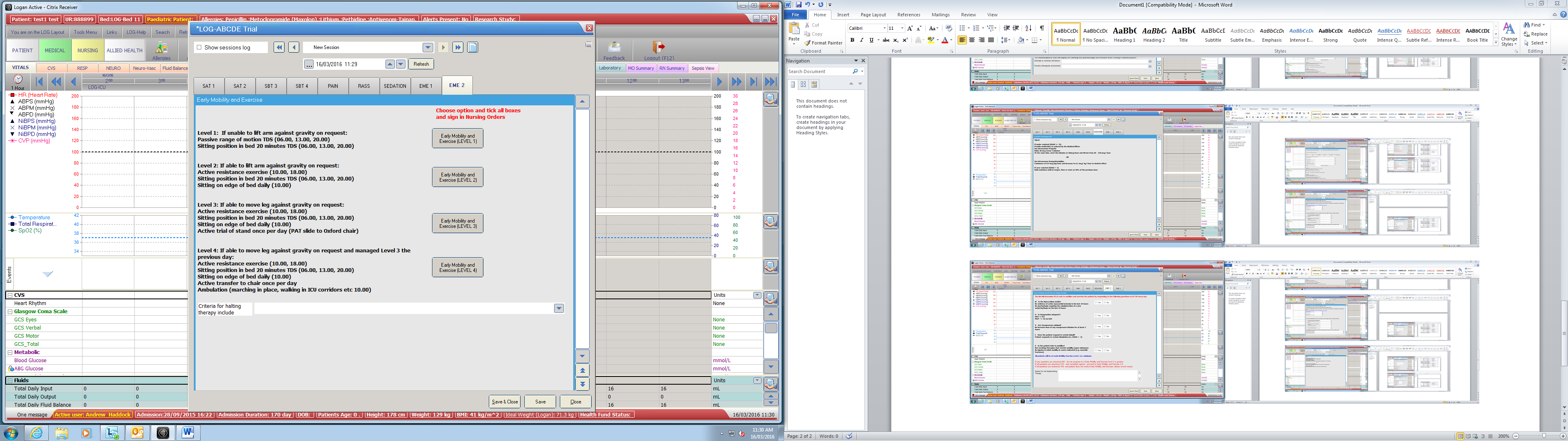


**Item 9. Tailoring: If the intervention was planned to be personalised, titrated or adapted, then describe what, why, when, and how**

Patients allocated to the intervention arm of the study received interventions (as described in Item 8) tailored to meet their clinical needs. Mandatory safety screens ensured that patients did not receive therapy that would cause harm (see Figure 1,3 and 4). Levels of exercise were tailored to the patient’s level of consciousness and ability (see Figure 5).

**Item 10. Modifications: If the intervention was modified during the course of the study, describe the changes (what, why, when, and how)**

There were no modifications made to the protocol during the course of the study.

**Item 11. How well (planned): If intervention adherence or fidelity was assessed, describe how and by whom, and if any strategies were used to maintain or improve fidelity, describe them**

Intervention adherence was a major outcome measure of this feasibility study. Adherence to the protocol was assessed on a daily basis by the research team. The protocol was scripted as a checklist and embedded in the ICU clinical information System. Paper based records were also available at the bed-side for staff to record reasons for not providing the interventions. Adherence to the protocol was assessed on a daily basis by the principle investigator and research team. Communication via newsletters, ward meetings, group and individual education sessions were provided to ensure and improve intervention fidelity.

**Item 12: How well (actual): If intervention adherence or fidelity was assessed, describe the extent to which the intervention was delivered as planned**

The prescribed exercise sessions were delivered as prescribed on 92% of ventilated days. A total of 432 exercise sessions were provided out of a total of 448 prescribed sessions. This amounts to 96.4% of total sessions provided over the course of the trial.
